# First bloodstream infection caused by *Prevotella copri* in a heart failure elderly patient with *Prevotella*-dominated gut microbiota: a case report

**DOI:** 10.1186/s13099-019-0325-6

**Published:** 2019-09-18

**Authors:** Patrizia Posteraro, Flavio De Maio, Giulia Menchinelli, Ivana Palucci, Federica Maria Errico, Mariantonietta Carbone, Maurizio Sanguinetti, Antonio Gasbarrini, Brunella Posteraro

**Affiliations:** 1 Laboratorio di Analisi Chimico-Cliniche e Microbiologiche, Ospedale San Carlo GVM, Rome, Italy; 2 Istituto di Microbiologia, Università Cattolica del Sacro Cuore, Rome, Italy; 3 Unità di Cardiologia, Ospedale San Carlo GVM, Rome, Italy; 4 Dipartimento di Scienze di Laboratorio e Infettivologiche, Fondazione Policlinico Universitario A. Gemelli IRCCS, Rome, Italy; 5 Istituto di Patologia Medica e Semeiotica Medica, Università Cattolica del Sacro Cuore, Rome, Italy; 6 Dipartimento di Scienze Gastroenterologiche, Endocrino-Metaboliche e Nefro-Urologiche, Fondazione Policlinico Universitario A. Gemelli IRCCS, Rome, Italy

**Keywords:** Bloodstream infection, *Prevotella* species, Microbiota, Heart failure, Precision medicine

## Abstract

**Background:**

Bloodstream infection (BSI) is a constant threat for hospitalized patients, and elderly patients are particularly susceptible to BSI caused by anaerobic bacteria. Changes in the gut microbiota composition may lead to pathogen overgrowth and translocation into the bloodstream. Consequently, domination of specific taxa in the intestinal bacterial community seems to be associated with a higher risk of bacteremia in some patient populations.

**Case presentation:**

Here, we report the case of a 90-year-old heart failure (HF) patient who was admitted to the hospital for an acute state of cardiac decompensation. Twenty days after admission, he was febrile to 38.2 **°**C whereas his white blood count and C-reactive protein increased to 6190 cells/μL and 31.2 mg/L, respectively. Of the patient’s blood culture (BC) bottle pairs collected under the suspicion of infection, the anaerobic bottle yielded an organism that was later identified as *Prevotella copri*. Concomitantly, the patient’s fecal sample was obtained for the intestinal microbiota characterization by sequencing the V3/V4/V6 regions of the bacterial 16S rRNA gene. The analysis revealed highest relative abundances of *Bacteroidales* (34.1%), *Prevotellaceae* (19.0%), *Prevotella* (15.2%), and *P. copri* (6.1%) taxa, indicating that the patient’s gut microbiota was dominated by *Prevotella* organisms. The patient was successfully treated with metronidazole, and was discharged to a long-term care facility at 35 days of admission.

**Conclusions:**

We provide the first evidence for a clinically significant BSI caused by *P. copri* and its relationship to a *Prevotella*-rich gut microbiota in the HF patient setting. When strengthening the pathogenicity of *P. copri*, the present case suggests that the gut may be a source of BSI caused by the rare anaerobic organism. Future studies are necessary to assess the role of the gut microbiota profiling for precise identification and targeted treatment of patients at high risk of BSI.

## Background

Bloodstream infection (BSI) is a constant menace for the hospitalized patients because of a number of risk factors related to underlying patient’s diseases and/or conditions [1]. In particular, factors as cardiovascular disease, liver cirrhosis, diabetes mellitus, and cancer, either alone or concomitant with immunosuppressive therapies and/or presence of indwelling devices, concern patients in internal medicine hospital wards [2]. Consequent to unprecedented population aging, the today’s patients are mostly elderly persons, and this makes their management particularly complex [3]. Since aged patients are at higher risk for infection because of more pronounced impairment of the anti-infective defenses, diagnosing BSI in such patients should require a high degree of suspicion [2, 3]. Although bacteremia has long recognized as a common disorder in the elderly [4], presentations of bacteremia are often atypical because older patients tend to have fewer symptoms compared to younger patients, and this may unfavorably influence the BSI outcomes [3].

Elderly patients seem to have a predilection for BSIs caused by anaerobic bacteria [4], with anaerobes accounting for 2–5% of BSIs in most studies [3]. *Bacteroides fragilis* is the most common blood isolate recovered from adult patients with anaerobic bacteremia, whereas other species include *Peptostreptococcus*, *Clostridium* spp., and *Fusobacterium* spp.; many of these infections are polymicrobial [5]. In a case series of *Peptoniphilus* spp. (i.e., Gram-positive anaerobic cocci formerly classified in the genus *Peptostreptococcus*) causing BSI in adults, three of 15 (20%) of the case patients died, and all of them were elderly (age range 82–96 years) with significant underlying comorbidities [6]. While seven (47%) of the 15 patients had polymicrobial BSIs, it is worthy to note that the recovery of *Peptoniphilus* alone, and not just as part of a polymicrobial infection, occurred in that study [6]. However, the clinical significance and the pathogenic potential of rare anaerobic bacteria in BSI remains undefined.

Besides having changed the clinicians’ ideas about microbes in human health and disease [7], intense research into the gut microbiota has shed light on how the intestinal microbial community affects the susceptibility to infectious diseases [8]. In this context, recent studies have shown that the increased relative abundance (also termed intestinal “domination”) of specific taxa in the gut bacterial communities (intestinal microbiota) enhanced the risk of bacteremia in some adult patient populations [9, 10]. Thus, unspecified factors would contribute to the microbiota compositional changes and drive pathogen (also termed “pathobiont”) overgrowth and translocation into the bloodstream [11]. Additionally, key association studies between specific changes in the intestinal microbiota composition and disease have shown that *Blautia*, *Collinsella*, unclassified *Erysipelotrichaceae*, and unclassified *Ruminococcaceae* significantly decreased in heart failure (HF) [12], whereas expansion of *Prevotella copri* correlated with predisposition to arthritis [13].

To our knowledge, the present report describes for the first time a symptomatic BSI caused by *P. copri* in a HF elderly patient, and relates this finding to the patient’s gut microbiota dominated by *Prevotella* organisms.

## Case presentation

A 90-year-old man was admitted to the Cardiology ward of the Ospedale San Carlo GVM of Rome, Italy, because of an acute state of cardiac decompensation. His presenting symptoms were weakness, shortness of breath, and peripheral edema and cyanosis. The patient had a long history of HF due to dilated cardiomyopathy, for which he had undergone to mitral valve repair. Three years before, he had a pacemaker implanted. The day of admission, on hospital day 1 (HD1), the echocardiography revealed that the mitral valve insufficiency was moderate whereas the tricuspid valve insufficiency was severe. The left ventricular ejection fraction (LVEF) was slightly reduced (52%). According to current HF treatment guidelines [14], the patient was undergoing a drug treatment with angiotensin-converting enzyme inhibitor, beta-blocker, diuretic agent, aldosterone antagonist, and antiarrhythmic/heart rate modulating agent. He received levothyroxine and pantoprazole as further medications. Consistent with a previously diagnosed chronic renal failure, the patient’s serum creatinine level was 2.48 mg/dL (reference range 0.70 to 1.20 mg/dL). Serum marker measurement tests revealed a C-reactive protein (CRP) of 43.2 mg/L (reference range 0.0 to < 5.0 mg/L) and an N-terminal of the prohormone brain natriuretic peptide (NT-proBNP) of 33,826 pg/mL (reference range 0.0 to < 700.0 pg/mL). A complete blood cell count test showed a white blood cell (WBC) count of 2830 cells/μL (reference range 4000 to 10,000 cells/μL). The electrocardiogram did reveal no substantial acute alterations. Based on these findings, the patient’s drug treatment remained unchanged. Fifteen days after admission, on HD16, the patient’s serum CRP and NT-proBNP levels decreased to 6.5 mg/L and 4761 pg/mL, respectively. Therefore, physicians planned the patient’s transfer to a long-term care facility.

Twenty days after admission, on HD21, the patient was found to be febrile to 38.2 °C, tachycardic, and tachypnic on physical exam. Laboratory tests revealed a WBC count of 6190 cells/μL, a CRP of 31.2 mg/L, and an erythrocyte sedimentation rate of 49.0 mm/h (reference range 0.0 to 12 mm/h). With the strong suspicion of infection, the patient’s blood and other body sites were sampled for microbiological cultures. After 72-h incubation of blood culture (BC) bottles’ (aerobic and anaerobic) pairs in the BACT/ALERT^®^ 3D automated BC system (bioMérieux, Marcy l’Étoile, France), a single anaerobic bottle from the patient’s BC set yielded a microorganism that appeared as a rod-shaped Gram-negative bacterium at the Gram stain microscopic observation. When subcultured on standard media, the bacterium grew only under anaerobic conditions. Pending the infecting-organism’s identification results (see below), the patient started on empirical antimicrobial therapy with piperacillin–tazobactam and metronidazole.

Initially, the MALDI BioTyper^®^ system (Bruker Daltonics, Bremen, Germany) based identification provided a *Prevotella stercorea* DSM 18206T organism as the third best match hit against the MALDI reference database, with an identification score value of 1.400 (not reliable identification score, ≤ 1.699; reliable identification score, > 1.699). The first and second match hits were *Paracoccus versutus* B352 UFL (score value, 1.505) and *Staphylococcus capitis* DSM 20325 (score value, 1.438). Then, the *Prevotella* organism was reliably identified as *P. copri* through comparing the partial 16S ribosomal RNA (rRNA) gene sequence (ABI Prism 3130 sequencer; Applied Biosystems, Foster City, CA) against the NCBI database using BLAST, that yielded > 99% identity with the *P. copri* sequence (GenBank accession, MG592701.1). In the meantime, we obtained a patient’s fecal sample for the 16S rRNA gene-targeted intestinal microbiota profiling (see Additional file 1: Methods). As shown in Table 1, sequencing of the V3, V4, and V6 regions of 16S rRNA gene showed highest relative abundances for the *Bacteroidales* order (34.1%), *Prevotellaceae* family (19.0%), *Prevotella* genus (15.2%), and *P. copri* species (6.1%). Interestingly, together with other *Prevotella* species (DJF RP53, BI-42, and DJF B112), *P. copri* accounted for 10.5% of all the intestinal microbiota composing (classified and unclassified) species. All these taxa belong to the *Bacteroidetes* phylum, which was the second most abundant (34.6%) after the *Firmicutes* phylum (37.0%) in the patient’s intestinal microbiota.

**Table 1:** Most relatively abundant bacteria on order, family, genus, and species level identified in the patient’s fecal sample

Taxa	% abundance	Taxa	% abundance
Order		Genus	
*Bacteroidales*	34.114	*Prevotella*	15.253
*Clostridiales*	27.590	*Faecalibacterium*	8.215
*Selenomonadales*	7.493	*Dialister*	6.372
		*Parabacteroides*	3.849
		*Bacteroides*	3.750
Family		Species	
*Prevotellaceae*	19.024	*Prevotella copri*	6.133
*Ruminococcaceae*	16.519	*Dialister succinatiphilus*	5.869
*Lachnospiraceae*	9.157	*Faecalibacterium prausnitzii*	3.180
*Porphyromonadaceae*	6.999	*Parabacteroides distasonis*	2.488
*Veillonellaceae*	6.758	*Prevotella* sp. (DJF RP53)	2.050
*Bacteroidaceae*	3.750		

The listed taxa belong to the *Firmicutes* and *Bacteroidetes* phyla, whose relative abundances were 37.054% and 34.664%, respectively. Unlisted species include two other *Prevotella* sp. (BI-42 and DJF B112), whose relative abundances were 1.504% and 0.836%, respectively

On HD26, the patient switched to metronidazole alone while subsequently collected BCs became negative. On HD35, he was hemodynamically stable and was finally discharged to a long-term care facility.

## Discussion and conclusions

The present case’s patient is the archetype of internal medicine patients in whom multiple comorbidities, polypharmacy, and length of hospitalization may concur to the acquisition of BSI [2]. With regard to HF (the main patient’s comorbidity), it should be recalled that the disease is not simply a cardiac disease but rather a complex multi-organ clinical syndrome [15]. In view of the new disease concepts [16], intestinal dysbiosis—a state of microbial community imbalance—in HF [12] could not only represent a key pathogenic factor but also mediate the leakage of the immune barrier, leading to chronic inflammatory disorders [17]. Until now, no studies have shown the potential of *P. copri* to break down the intestinal barrier [18], which may indeed trigger systemic inflammation through the microbial or endotoxin translocation to the systemic circulation in decompensated HF patients [19].

Here, we provided the first evidence that a *Prevotella*-rich intestinal microbiota relates to the onset of a clinically significant BSI caused by *P. copri* in the HF patient setting, thereby supporting the growing emphasis on *P. copri*—and *Prevotella* species in general—as a pathobiont but not as a beneficial bacterium [20–22]. The relative abundance observed for *Prevotellaceae* in our patient is lower than the relative abundance percentages reported as threshold values for the increased risk of *Enterococcaceae*, *Streptococcaceae*, and *Proteobacteria* bacteremia in hematopoietic stem cell transplantation or long-term acute care hospital patients [9, 10]. Unlike in our case, patients in the two studies have common risk factors for bacterial translocation in the gut [9, 10], and one study apply a threshold of 22% relative abundance to predict bacteremia caused by *Klebsiella pneumoniae* carbapenemase-producing *Klebsiella pneumoniae* (KPC-Kp) [10]. Additionally, association between exposure to certain antibiotics (i.e., those more strongly associated with clinically important microbiota disruption) and subsequent sepsis within 90 days after a hospital stay has recently been documented [23]. In our case, the patient did not receive antibiotics during the hospital stay before the BSI onset nor have any of predisposing factors to anaerobic bacteremia, which include malignant neoplasms, hematologic disorders, organ transplantation, diabetes mellitus, and an undrained abscess among the others [5]. Therefore, it is plausible that the occupation of at least 15% of our patient’s fecal microbiota by *Prevotella* as a single predominating bacterial taxon would have allowed *P. copri* to cross the intestinal barrier and then became detectable in the patient’s blood. Yet, it is unclear if the long-term administration of proton-pump inhibitor could have altered the gut microbiota composition in our patient. In accordance with us, *Prevotella* had a relative abundance between 10 and 40% in 16% of microbiota samples analyzed in one study, but 75% of samples had a relative abundance less than 5% [24]. Because differences in the samples’ *Prevotella* (and *Bacteroides*) abundances did not represent consistent microbial communities within the “enterotypes” described originally [20], Gorvitovskaia et al. proposed to use “biomarker” for representing the dominant taxon of a given microbial community [20]. Thus, we can interpret *Prevotella* not only as a “biomarker” of diet or lifestyle [20] but also of disease state.

This case shows that physicians should suspect anaerobes as the cause of bacteremia not only based on typical clinical predictors [5] and, especially in patients with complex underlying disease, should require BCs supporting the growth of anaerobic bacteria always and when not only an anaerobic infection is present or suspected clinically [5]. Like other Gram-negative bacilli, *Prevotella* species are non-spore-forming, non-motile, singular cells that thrive under anaerobic growth conditions. Therefore, their isolation from the patient’s BCs—the today’s laboratory diagnostic standard for BSI—is somewhat difficult because of prolonged organism’s growth times that often exceed the conventional 5-day incubation BC period [25]. Our patient presented with fever (> 38 °C) which represents a typical symptom of bacteremia [3, 5]. By reviewing studies that evaluated aged (65 years and older) patients hospitalized with BSIs, Yahav et al. [3] reported that the fever was present in at least 75%, whereas leukocytosis in 39–73% and leukopenia in ~ 10%, of the patients studied. In one of these studies, elevation of CRP was as common in older patients as in younger patients [26]. In our patient, increased levels of either WBC (twofold) or CRP (fivefold) occurred immediately prior to the BSI onset. This strengthens the pathogenicity of the *P. copri* isolate and, importantly, counterbalances the fact we were unable to recover the isolate from more than one blood sample of our patient, which is often the case in many instances [5]. Consistent with the fact that anaerobic bacteria including *Prevotella* species are slow and/or fastidious growing on standard subculture media, we could not obtain reliable identification of the patient’s isolate with the MALDI BioTyper^®^ system, which is a well-suited method in routine laboratories [27]. As anaerobic Gram-negative isolates can be resistant to penicillin, a combination of a beta-lactamase inhibitor and a penicillin may be appropriate in cases of infection caused by *B. fragilis* group, *Fusobacterium* species, or pigmented *Prevotella* species [5]. Thus, based on the positivity of the patient’s anaerobic BC results physicians selected a combination of piperacillin–tazobactam and metronidazole to treat empirically the patient. Following the availability of the isolate’s final identification by the PCR sequencing method, physicians shifted the patient’s antimicrobial treatment to only metronidazole, and this treatment was successful.

In conclusion, we documented the first clinically significant recovery of *P. copri* from the blood of a HF patient. At the same time, we detected *P. copri* as the relatively most abundant organism in the gut of the patient, which strongly indicates a relationship between *P. copri* as a cause of primary BSI and an intestinal microbiota dominated by the same organism. Although we did not show that intestinal dominance by the BSI pathogen preceded the onset of infection [28], the assumption of a higher relative abundance of *P. copri* in the patient’s gut prior to infection is reasonable. Finally, our case underlines the importance of profiling the gut microbiota in patients particularly at risk of BSI to allow a precise identification and targeted treatment of these patients. In this context, the metronidazole prophylaxis to cardiac patients undergoing any interventions would be extended to routine administration on hospitalization if their gut microbiota was proven to be altered.

## Supplementary information

**Additional file 1.** Methods for the 16 rRNA gene based intestinal microbiota characterization.

## Data Availability

The partial 16S rRNA gene sequence of the patient’s isolate is available in the GenBank database under the accession number MK680126. The datasets analyzed during this study are available in the NCBI Sequence Read Archive repository [PRJNA529053] (https://www.ncbi.nlm.gov/bioproject/529053).

## Abbreviations

BSI: bloodstream infection
HF: heart failure
HD: hospital day
LVEF: left ventricular ejection fraction
CRP: C-reactive protein
NT-proBNP: N-terminal of the prohormone brain natriuretic peptide
WBC: white blood cell
BC: blood culture
MALDI: matrix-assisted laser desorption ionization
rRNA: ribosomal RNA
