# Supplementary material for: First bloodstream infection caused by Prevotella copri in a heart failure elderly patient with Prevotella-dominated gut microbiota: a case report
Source: Gut Pathog. 2019 Sep 18;11:44. doi: 10.1186/s13099-019-0325-6 (PMC6749625; doi:10.1186/s13099-019-0325-6)
Supplement: Supplementary file 1 — Additional file 1. Methods for the 16 rRNA gene based intestinal microbiota characterization. [file 13099_2019_325_MOESM1_ESM.docx]

**Additional file 1: Methods for the 16 rRNA gene based intestinal microbiota characterization**

**DNA extraction and sequencing**

A patient’s fecal sample was collected in a standard stool collection tube and transported in a refrigerated package to the Microbiology Laboratory of the Fondazione Policlinico A. Gemelli IRCCS of Rome, Italy. Upon arrival, the sample was immediately processed for the DNA extraction using the **QIAamp^®^ DNA Stool Mini kit (**Qiagen, Hilden, Germany**). Extracted DNA was** PCR amplified with dual-index primers targeting the V3, V4, and V6 regions of the bacterial 16S rRNA gene, using the ARROWforNGS Microbiota solution B kit (ARROW Diagnostics, Genova, Italy; catalog no. AD-002.024). A control sterile tube sample that had undergone the same DNA extraction and PCR amplification procedures was included [1]. Before sequencing, amplicons were purified using the Agencourt^®^ AMPure XP PCR purification system (Beckman Coulter, Milan, Italy), and equal amounts (10 nM) of sample’s DNA were pooled and diluted to reach the 4-nM concentration, as appropriate. Finally, an 8-pM denatured DNA sample was used to generate sequences using the 300 cycle MiSeq Reagent Nano kit v2 (Illumina, San Diego, CA; catalog no. MS-103-1001) on an Illumina^®^ MiSeq^®^ sequencer (2 × 250 bp).

**Sequence data analysis**

Sequence data were analyzed using the MicrobAT system [2], a custom bioinformatics pipeline developed by SmartSeq S.r.l. (Novara, Italy). During MicrobAT processing, demultiplexed sequences with reads of length less than 200 nucleotides, an average Phred quality score below 25, and an at least one ambiguous base were discarded. The resulting 56,961 patient’s sample sequences were aligned at a 97% sequence similarity and assigned to taxonomic (e.g., species) levels at an 80% classification threshold, using the Ribosomal Database Project (RDP) classifier (release 11.5) [3]. Species that did not meet these criteria were assigned to the corresponding group named “unclassified [genus]”. Sequence data for the patient’s sample was deposited in the BioProject PRJNA529053.

**References**

1. Bik EM. The hoops, hopes, and hypes of human microbiome research. Yale J Biol Med. 2016;89:363–73.
2. Novello G, Gamalero E, Bona E, Boatti L, Mignone F, Massa N, Cesaro P, Lingua G, Berta G. The rhizosphere bacterial microbiota of *Vitis vinifera* cv. Pinot Noir in an integrated pest management vineyard. Front Microbiol. 2017;8:1528.
3. Cole JR, Wang Q, Fish JA, Chai B, McGarrell DM, Sun Y, Brown CT, Porras-Alfaro A, Kuske CR, Tiedje JM. Ribosomal Database Project: data and tools for high throughput rRNA analysis. Nucleic Acids Res. 2014;42(Database issue):D633–D42.
